# Association of Pregnancy Characteristics and Maternal Mortality With Amniotic Fluid Embolism

**DOI:** 10.1001/jamanetworkopen.2022.42842

**Published:** 2022-11-18

**Authors:** Genevieve R. Mazza, Ariane C. Youssefzadeh, Maximilian Klar, Mirjam Kunze, Shinya Matsuzaki, Rachel S. Mandelbaum, Joseph G. Ouzounian, Koji Matsuo

**Affiliations:** 1 Division of Gynecologic Oncology, Department of Obstetrics and Gynecology, University of Southern California, Los Angeles; 2 Department of Obstetrics and Gynecology, University of Freiburg Faculty of Medicine, Freiburg, Germany; 3 Department of Gynecology, Osaka International Cancer Institute, Osaka, Japan; 4 Division of Reproductive Endocrinology & Infertility, Department of Obstetrics and Gynecology, University of Southern California, Los Angeles; 5 Division of Maternal-Fetal Medicine, Department of Obstetrics and Gynecology, University of Southern California, Los Angeles; 6 Norris Comprehensive Cancer Center, University of Southern California, Los Angeles

## Abstract

**Importance:**

Amniotic fluid embolism (AFE) is an uncommon pregnancy complication but is associated with high maternal mortality. Because of the rarity of AFE, associated risks factors and maternal outcomes have been relatively understudied.

**Objective:**

To examine the clinical, pregnancy, and delivery characteristics and the maternal outcomes related to AFE in a recent period in the US.

**Design, Setting, and Participants:**

This retrospective cohort study examined hospital deliveries from January 1, 2016, to December 31, 2019, from the Healthcare Cost and Utilization Project’s National Inpatient Sample.

**Main Outcomes and Measures:**

The primary outcome was clinical, pregnancy, and delivery characteristics of AFE, assessed with a multivariable binary logistic regression model. The coprimary outcome was failure to rescue, defined as maternal mortality after AFE. Associations with other severe maternal morbidity indicators and failure to rescue per clinical and pregnancy characteristics were also assessed.

**Results:**

A total of 14 684 135 deliveries were examined, with AFE diagnosed in 880 women, corresponding to an incidence rate of 6.0 per 100 000 deliveries. The cohort-level median patient age was 29 years (IQR, 25-33 years). In a multivariable analysis, (1) patient factors of older age, Asian and Black race, Western US region, pregestational hypertension, asthma, illicit substance use, and grand multiparity; (2) pregnancy factors of placental accreta spectrum (PAS), placental abruption, uterine rupture, polyhydramnios, chorioamnionitis, preeclampsia, fetal growth restriction, and fetal demise; and (3) delivery factors of early gestational age, cervical ripening, cesarean delivery, operative delivery, and manual removal were associated with AFE. Among these characteristics, PAS had the largest association with AFE (adjusted odds ratio [aOR], 10.01; 95% CI, 7.03-14.24). When stratified by the PAS subtypes, more severe forms of PAS had a greater association with AFE (aOR for increta and percreta, 17.35; 95% CI, 10.21-28.48; and aOR for accreta, 7.62; 95% CI, 4.83-12.01). Patients who had AFE were more likely to have coagulopathy (aOR, 24.68; 95% CI, 19.38-31.44), cardiac arrest (aOR, 24.56; 95% CI, 17.84-33.81), and adult respiratory distress syndrome (aOR, 10.72; 95% CI, 8.09-14.20). The failure-to-rescue rate after AFE was 17.0% overall. However, the failure-to-rescue rate exceeded 30% when AFE co-occurred with other severe maternal morbidity indicators: 45.8% for AFE, cardiac arrest, and coagulopathy; 43.2% for AFE, shock, and cardiac rhythm conversion; and 38.6% for AFE, cardiac arrest, coagulopathy, and shock. The failure-to-rescue rate after AFE also exceeded 30% when AFE occurred in the setting of placental pathology: 42.9% for AFE and PAS and 31.3% for AFE and placental abruption.

**Conclusions and Relevance:**

This contemporaneous, national-level analysis validated previously known risk factors for AFE and confirmed the dismal outcomes of pregnancy complicated by AFE. The association between PAS and AFE, which was not previously reported, warrants further investigation.

## Key Points

**Question:**

What pregnancy characteristics and maternal outcomes are associated with amniotic fluid embolism (AFE)?

**Findings:**

In this cohort study of 14.6 million deliveries in the National Inpatient Sample from 2016 to 2019, the incidence of AFE was 6.0 per 100 000 deliveries, and placental accreta spectrum was associated with a 10-times increased risk of AFE. Amniotic fluid embolism was associated with other severe maternal morbidities, including coagulopathy, cardiac arrest, and adult respiratory distress syndrome; failure-to-rescue rate after AFE was 17% overall but could exceed 30% in the setting of placental pathology or other comorbidities.

**Meaning:**

This national-level analysis confirmed the dismal outcomes of pregnancies complicated by AFE and suggests that placental accreta spectrum may be associated with AFE.

## Teaser

This cohort study of 14.6 million deliveries in the National Inpatient Sample from 2016 to 2019 examines the associated risks factors and maternal outcomes of amniotic fluid embolism.

## Introduction

Maternal mortality has more than doubled during the past 3 decades in the US, with most recent national estimates of 17.3 maternal deaths per 100 000 live births in 2017.^1^ Amniotic fluid embolism (AFE), despite its rarity, is a leading cause of maternal mortality that often presents as sudden cardiovascular collapse, respiratory distress, and coagulopathy.^1,2,3^ The reported incidence ranges from 0.8 to 7.7 per 100 000 births, with similarly wide reports of case mortality ranging from 11% to 43%.^2,4,5,6,7,8,9,10^ These wide ranges are likely attributable to the lack of uniform diagnostic criteria.^6,11,12^ Recent evidence also suggests at least one-fifth of all cases of AFE were most likely incorrectly diagnosed (possible overdiagnosis), underscoring the diagnostic challenges surrounding AFE.^13^

Amniotic fluid embolism was first reported nearly a century ago and thought to be secondary to fetal debris occluding maternal pulmonary circulation.^2,14,15^ However, this assumption has been challenged for a variety of reasons, including the lack of evidence of pulmonary vessel obstruction in many cases of AFE.^2,14,15^ One plausible alternative hypothesis includes maternal exposure to fetal antigen that triggers complex physiologic derangements, including the activation of proinflammatory mediator systems.^2,11^ However, the exact cause of AFE remains unknown.^2,11^

Although the exact pathophysiologic mechanism is unknown, several risk factors are identified for AFE based on maternal, fetal, placental, and delivery factors.^2,4,5,6,7,8,9,10^ Continued validation and exploration of risk factors for AFE may lead to a better understanding of AFE pathophysiology. Furthermore, there is a paucity of recent US data on maternal outcomes after AFE. Thus, the objective of the current study was to examine the pregnancy characteristics and maternal outcomes related to AFE from 2016 to 2019 in the US.

## Methods

### Data

This retrospective cohort study used the National Inpatient Sample (NIS), which is distributed as part of the Healthcare Cost and Utilization Project by the Agency for Healthcare Research and Quality.^16^ The NIS represents hospital discharge data for more than 90% of the US population using survey weights based on a random sampling of 20% of hospitalized patients annually. The data set is both publicly available and deidentified; thus, this study was deemed exempt by the University of Southern California Institutional Review Board. This study followed the Strengthening the Reporting of Observational Studies in Epidemiology (STROBE) reporting guideline.^17^

### Study Population

The study population was patients who had a vaginal or cesarean delivery between January 1, 2016, and December 31, 2019. This study period was chosen to correspond with the introduction of *International Statistical Classification of Diseases and Related Health Problems, Tenth Revision* (*ICD-10*) codes in the NIS program and to assess the complete year. Case identification for vaginal and cesarean delivery was determined per the *International Statistical Classification of Diseases, Tenth Revision, Clinical Modification* (*ICD-10-CM*) and *ICD-10* Procedure Classification System (*ICD-10-PCS*) codes and the diagnosis-related group codes (eTable 1 in the Supplement).

### Outcome Measures

The main outcome measure was diagnosis of AFE. This study followed the Centers for Disease Control and Prevention (CDC) definition,^18^ and the *ICD-10* code of O88.1 was used to identify the patients who had AFE. Of note, the code does not specify the exact diagnostic criteria for AFE. Co-outcome measure was failure to rescue after AFE. Definition of failure to rescue followed prior studies^10,19^ and was determined as the maternal mortality rate among the patients who had AFE. The NIS program captures the mortality event that occurred during the index admission in the discrete variable.

### Clinical Information

The covariates examined in this study were selected based on prior studies^2,4,5,6,8,9,10,11,13,15^ for AFE (a total of 40 covariates): (1) patient demographic characteristics, (2) pregnancy characteristics, (3) hospital parameters, and (4) delivery data, including severe maternal morbidity (SMM). The *ICD-10* codes for the measured covariates were consistent during the study period (eTable 1 in the Supplement).

Patient baseline demographic characteristics included age (<25, 25-29, 30-34, 35-39, and ≥40 years), year of delivery (2016, 2017, 2018, and 2019), race and ethnicity (Asian, including Pacific Islander; Black; Hispanic; White; and other [including American Indian] per the NIS designation) as determined by the NIS program, primary expected payer (Medicaid; private, including health maintenance organization; Medicare; self-pay; and others), census-level median household income (every quartile), obesity, allergic spectrum (asthma, atopic dermatitis, and allergy status), substance use (tobacco, illicit drug, and alcohol), and grand multiparity. Race and ethnicity were evaluated because this information is associated with pregnancy characteristics and outcomes.

Pregnancy characteristics included maternal factors (diabetes [gestational or pregestational] and hypertensive disorders [gestational, pregestational, and preeclampsia]), placental pathology factors (placental previa, placenta accreta spectrum [PAS], placental abruption, and placenta malformation), fetal factors (multifetal gestation, breech presentation, polyhydramnios, oligohydramnios, fetal growth restriction, large for gestational age, fetal anomaly, and fetal demise), uterine factors (prior uterine scar, uterine myoma, and uterine rupture), and membranous factors (premature rupture of membrane [preterm or term] and chorioamnionitis).

Hospital information included bed capacity (small, medium, or large), hospital location and teaching status (rural, urban nonteaching, or urban teaching), and hospital region in the US (Northeast, Midwest, South, or West).

Delivery data included gestational age at delivery (≥39, 37-38 6/7, 34-36 6/7, or <34 weeks), induction of labor,^20^ mode of delivery (vaginal, operative delivery including vacuum-assisted and forceps, or cesarean), manual removal of placenta, and SMM other than AFE per the CDC definition for a total of 20 indicators (acute myocardial infarction, aneurysm, acute renal failure, adult respiratory distress syndrome, cardiac arrest or ventricular fibrillation, cardiac rhythm conversion, disseminated intravascular coagulation, eclampsia, heart failure or arrest during surgery or procedure, puerperal cerebrovascular disorders, pulmonary edema or acute heart failure, severe anesthesia complications, sepsis, shock, sickle cell disease with crisis, air and thrombotic embolism, blood products transfusion, hysterectomy, temporary tracheostomy, and ventilation).^18^

### Statistical Analysis

The first step of the analysis was to identify the independent characteristics associated with AFE at delivery. A binary logistic regression model was used for multivariable analysis. The initial selection criteria were *P* < .05 in univariable analysis, and a parsimonious, conditional backward-selection method was used with the stopping rule of *P* < .05 in the final model.^21^ This analytic approach was preplanned because of the assumption that the number of AFEs is likely small. Multicollinearity was assessed among covariates. Effect size for AFE was expressed with an adjusted odds ratio (aOR), corresponding to a 95% CI.

The next step of analysis was to assess the failure-to-rescue rate (mortality after AFE). First, the failure-to-rescue rate was examined in each SMM indicator, including AFE, in a bivariate fashion. Second, other SMM indicators independently associated with AFE were assessed with a multivariable binary logistic regression model (conditional backward selection). Failure-to-rescue rates were then assessed based on the combination pattern of AFE and other SMM indicators. A recursive partitioning analysis with χ^2^ automatic interaction detector method with a stopping rule of a maximum of 3 layers was used to identify the unique combination patterns to other SMM indicators.^22^

Several sensitivity analyses were undertaken to assess the robustness of the analytic findings. First, a classification tree was constructed to examine the incidence of AFE per the clinical and pregnancy characteristics. All the independent characteristics associated with AFE were entered into the model. Second, the failure-to-rescue rate associated with AFE was assessed per the clinical and pregnancy characteristics. Specifically, the outcomes were assessed in the setting of placental pathology (PAS and placental abruption) and per maternal age. These factors were selected in a post hoc approach. Third, PAS subtypes were assessed (accreta, increta, or percreta).

Fourth, the association between PAS and postpartum hemorrhage was assessed. This assessment was based on the rationale that although postpartum hemorrhage is not a CDC-defined SMM, this metric represents a key delivery outcome. Fifth, the delivery factors were excluded in the risk factor analysis. Sixth, cohort-level trend analysis was performed to examine the time-specific change in the at-risk groups for AFE (PAS, placental abruption, and older maternal age), assessed with the Cochran-Armitage trend test. This analysis was exploratory, and these pregnancy and prepregnancy factors were selected based on the post hoc observation.

The weighted values for national estimates provided by the NIS program were used for analysis, and statistical interpretation was based on a 2-tailed hypothesis. A *P* < .05 was considered statistically significant. Cases with missing information were grouped as 1 category in each variable. SPSS statistical software, version 28.0 (IBM Inc) was used for all analyses.

## Results

### Characteristics of AFE

A total of 14 684 135 deliveries for national estimates were examined for analysis, and 880 cases of AFE were recorded during the study period. This statistic makes the incidence rate of AFE 6.0 per 100 000 pregnancies (1 in 16 646 deliveries). The incidence of AFE only slightly increased from 5.2 to 6.5 per 100 000 deliveries from 2016 to 2019 (*P* = .04 for trend) (eFigure, A in the Supplement). The measured study covariates are given in eTable 2 in the Supplement. The cohort-level median patient age was 29 years (IQR, 25-33 years). Overall, of all the women, 6.0% were Asian, 14.4% were Black, 19.9% were Hispanic, 50.4% were White, 5.1% were of other races, and 4.3% had unknown race. A total of 51.3% were privately insured, and most patients delivered vaginally (63.8%), at 39 weeks’ gestation or later (62.0%), and at urban teaching centers (69.9%).

In a multivariable analysis, (1) patient factors of older age, Asian and Black race, Western US region, pregestational hypertension, asthma, illicit substance use, and grand multiparity; (2) pregnancy factors of PAS, placental abruption, uterine rupture, polyhydramnios, chorioamnionitis, preeclampsia, fetal growth restriction, and fetal demise; and (3) delivery factors of early gestational age, cervical ripening, cesarean delivery, operative delivery, and manual removal were associated with AFE (Table 1). These patient and pregnancy factors remained robust when delivery factors were excluded.

**Table 1.** Multivariable Analysis for Amniotic Fluid Embolism^a^

Characteristic	aOR (95%CI)	*P* value
Age, y		
<25	1 [Reference]	NA
25-29	1.14 (0.92-1.42)	.24
30-34	1.45 (1.17-1.79)	<.001
35-39	1.57 (1.24-1.98)	<.001
≥40	2.48 (1.86-3.31)	<.001
Unknown	NA	>.99
Race and ethnicity		
Asian	1.83 (1.44-2.31)	<.001
Black	1.41 (1.17-1.70)	<.001
Hispanic	0.79 (0.64-0.98)	.03
White	1 [Reference]	NA
Other^b^	1.10 (0.81-1.51)	.54
Unknown	1.45 (1.06-1.97)	.02
Primary expected payer		
Medicaid	1 [Reference]	NA
Private including HMO	0.82 (0.70-0.95)	.009
Medicare	1.32 (0.79-2.22)	.29
Self-pay	0.55 (0.33-0.93)	.02
Others	1.34 (0.94-1.90)	.10
Unknown	NA	.97
Asthma		
No	1 [Reference]	NA
Yes	1.59 (1.27-2.00)	<.001
Grand multiparity		
No	1 [Reference]	NA
Yes	2.41 (1.29-4.52)	.006
Illicit substance use		
No	1 [Reference]	NA
Yes	1.47 (1.09-1.98)	.01
Hypertensive disorder		
No	1 [Reference]	NA
Pregestational	1.38 (1.08-1.76)	.009
Gestational	0.74 (0.52-1.04)	.08
Preeclampsia	1.30 (1.03-1.63)	.03
Placental abruption		
No	1 [Reference]	NA
Yes	4.06 (3.17-5.21)	<.001
Placenta accreta spectrum		
No	1 [Reference]	NA
Yes	10.01 (7.03-14.24)	<.001
Uterine rupture		
No	1 [Reference]	NA
Yes	3.91 (2.07-7.36)	<.001
Fetal growth restriction		
No	1 [Reference]	NA
Yes	1.37 (1.05-1.79)	.02
Intrauterine fetal demise		
No	1 [Reference]	NA
Yes	3.32 (2.30-4.79)	<.001
Chorioamnionitis		
No	1 [Reference]	NA
Yes	1.64 (1.21-2.22)	<.001
Polyhydramnios		
No	1 [Reference]	NA
Yes	3.56 (2.76-4.60)	<.001
Gestational age at delivery, wk		
≥39	1 [Reference]	NA
37-38 6/7	0.96 (0.80-1.14)	.62
34-36 6/7	1.11 (0.88-1.41)	.38
<34	1.38 (1.06-1.80)	.02
Unknown	9.81 (7.76-12.38)	<.001
Induction or ripening		
No	1 [Reference]	NA
Yes	2.19 (1.68-2.86)	<.001
Delivery type		
Vaginal	1 [Reference]	NA
Forceps	3.17 (1.30-7.74)	.011
Vacuum assisted	4.95 (3.55-6.88)	<.001
Both	n/a	.99
Cesarean	6.41 (5.38-7.64)	<.001
Manual removal		
No	1 [Reference]	NA
Yes	4.82 (2.86-8.14)	<.001
Hospital region		
Northeast	1 [Reference]	NA
Midwest	1.01 (0.79-1.30)	.92
South	1.18 (0.95-1.45)	.13
West	1.79 (1.44-2.22)	<.001

Abbreviations: aOR, adjusted odds ratio; HMO, health maintenance organization; NA, not applicable.

^a^ A binary logistic regression model was used for multivariable analysis. Initial covariate selection was *P* < .05 in univariable analysis. A parsimonious, conditional backward method was used for the final modeling.

^b^ Including American Indian (as designated by the National Inpatient Sample) grouped by the program.

Among these independent characteristics, PAS had the greatest association with AFE (aOR, 10.01; 95% CI, 7.03-14.24) (Table 1 and eTable 3 in the Supplement). Among the patients with AFE, 4.0% had a diagnosis of PAS. When stratified by PAS subtypes, more severe forms of PAS had a greater association with AFE (aOR for increta and percreta, 17.35; 95% CI, 10.21-29.48; and aOR for accreta, 7.62; 95% CI, 4.83-12.01).

A 3-layer classification tree model identified 14 unique patterns for AFE (eTable 4 in the Supplement). The first layer allocator was PAS (192.4 vs 5.8 per 100 000 deliveries, *P* < .001), followed by placental abruption and gestational age in the second layer allocation. The most common group was term vaginal delivery (57.8%), with the incidence of AFE being 1.2 per 100 000 deliveries. Patients who had PAS, placental abruption, and late-preterm to early-term delivery had the highest incidence rate of AFE.

### Failure to Rescue After AFE

**Figure 1.**
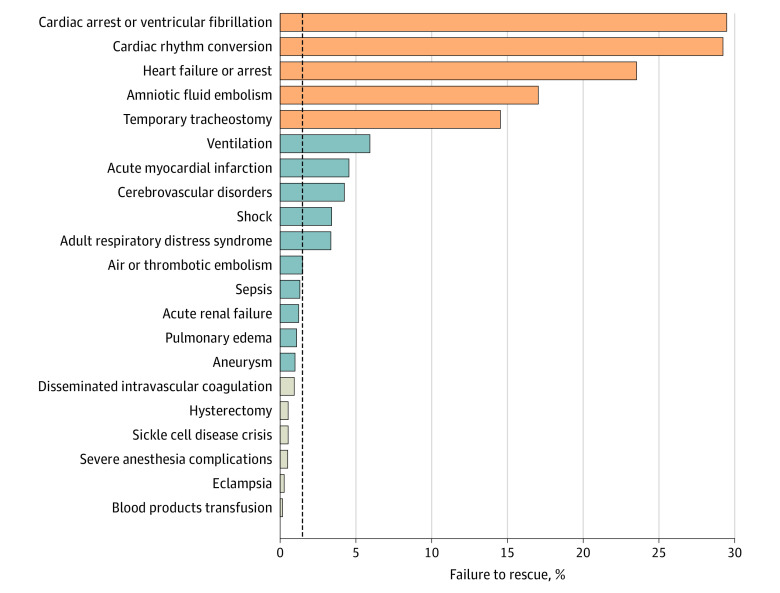
Failure to Rescue per Severe Maternal Morbidity Indicator The failure-to-rescue rate is shown for each severe maternal morbidity indicator, including amniotic fluid embolism (a total of 21 indicators defined by the Centers for Disease Control and Prevention). Failure to rescue was defined by mortality after the morbidity event. The vertical dashed line indicates the failure-to-rescue rate of 1.5%, which is the median value of 21 indicators. Orange bars prepresent a failure-to-rescue rate of 10% or greater; blue bars, a rate of at least 1% but less than 10%; and gray bars, a rate of less than 10%.

Crude failure-to-rescue rates of 21 SMM indicators, including AFE, are shown in Figure 1. The median failure-to-rescue rate among 21 indicators was 1.5% (range, 0.2%-29.5%). Of those, the failure-to-rescue rate exceeded 10% in 5 indicators, with cardiac arrest having the highest rate (29.5%). The failure-to-rescue rate after AFE was 17.0% (fourth highest indicator). Among the 150 patients who had a fatal outcome after AFE, 110 (73.3%) had the death event within 1 day of hospital admission. Postpartum hemorrhage occurred in nearly half of AFE cases (49.4% vs 4.4%; *P* < .001).

**Figure 2.**
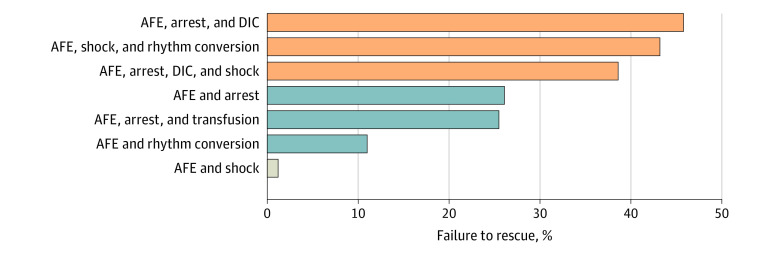
Failure to Rescue After Amniotic Fluid Embolism (AFE) With the Addition of a Morbidity Indicator Failure-to-rescue rates after AFE are shown based on the addition of other severe maternal morbidity indicators, determined by a classification tree model analysis. Orange bars represent a failure-to-rescue rate of 30% or greater; blue bars, a rate of at least 10% but less than 30%; and gray bars, a rate of less than 10%. DIC indicates disseminated intravascular coagulation.

Among the SMM indicators, 8 indicators were independently associated with AFE (Table 2). Of those, coagulopathy (aOR, 24.68; 95% CI, 19.38-31.44), cardiac arrest (aOR, 24.56; 95% CI, 17.84-33.81), and adult respiratory distress syndrome (aOR, 10.72; 95% CI, 8.09-14.20) had the most marked association with AFE (aOR > 10). A 3-layer classification tree with these 8 independent SMM indicators for AFE identified 7 unique morbidity patterns (Figure 2). The failure-to-rescue rate exceeded 30% when AFE occurred with other SMM indicators: 45.8% for AFE, cardiac arrest, and coagulopathy; 43.2% for AFE, shock, and cardiac rhythm conversion; and 38.6% for AFE, cardiac arrest, coagulopathy, and shock.

**Table 2.** Association Between Amniotic Fluid Embolism and Other Severe Maternal Morbidity Indicators^a^

Morbidity indicator	aOR (95%CI)	*P* value
Disseminated intravascular coagulation	24.68 (19.38-31.44)	<.001
Cardiac arrest/ventricular fibrillation	24.56 (17.84-33.81)	<.001
Adult respiratory distress syndrome	10.72 (8.09-14.20)	<.001
Cardiac rhythm conversion	8.59 (6.15-12.01)	<.001
Shock	4.51 (3.38-6.04)	<.001
Heart failure/arrest	3.25 (1.76-5.99)	<.001
Blood products transfusion	2.96 (2.34-3.75)	<.001
Eclampsia	2.96 (1.90-4.62)	<.001

Abbreviation: aOR, adjusted odds ratio.

^a^ A multivariable binary logistic regression model with parsimonious, conditional backward selection was used for analysis. The effect size indicates the odds of other morbidity indicator with the presence of amniotic fluid embolism.

**Figure 3.**
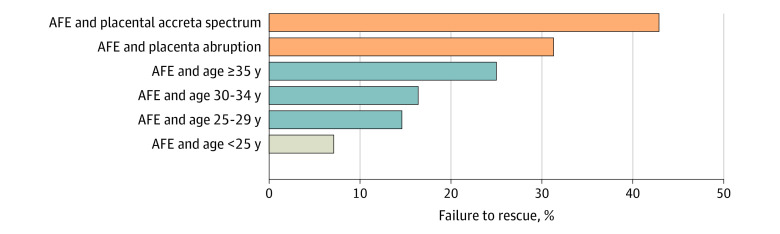
Failure to Rescue After Amniotic Fluid Embolism (AFE) per Clinical or Pregnancy Factors Failure-to-rescue rates after AFE are shown based on the presence of placental pathology or per age stratification. Orange bars represent a failure-to-rescue rate of 30% or greater; blue bars, a rate of at least 10% but less than 30%; and gray bar, a rate of less than 10%.

Failure to rescue after AFE also exceeded 30% when AFE occurred in the setting of placental pathology: 42.9% for AFE and PAS and 31.3% for AFE and placenta abruption (Figure 3). Among patients who had AFE, older age was associated with a higher failure-to-rescue rate, ranging from 7.1% for those younger than 25 years to 25.0% for those 35 years or older.

### Cohort-Level Trends of At-Risk Groups

At-risk groups for AFE gradually increased during the study period: PAS, from 1.17 to 1.29 per 1000 deliveries (11.0% relative increase); placental abruption, from 10.9 to 11.3 per 1000 deliveries (3.7% relative increase); and age 40 years or older, from 30.7 to 34.2 per 1000 deliveries (11.4% relative increase) (*P* < .001 for trend for all) (eFigure, B-D in the Supplement).

## Discussion

### Principal Findings

The key results of the current study are as follows. First, placental pathology, specifically PAS, was strongly associated with AFE at the time of delivery. Second, failure to rescue after AFE was strikingly high but varied based on other clinical factors.

### Risk Factors

The association of PAS with AFE was unexpected because this observation was not reported previously. The findings here corroborate the results of prior studies^2,5,9,10,13,15^ that demonstrated an association between placental pathology, such as placenta previa and placental abruption, with AFE. However, the association between PAS and AFE found in this study adds new information to the literature and warrants particular attention.

In PAS, the absence of the normal decidua basalis leads to direct trophoblast attachment to and invasion through the myometrium.^23^ One possible explanation for the association between PAS and AFE is that the disruption of the maternal-fetal interface may result in increased fetal antigen exposure and/or permit amniotic fluid to enter the maternal circulation.^2,24^ The severity of PAS was associated with AFE, suggesting a linear relationship between the degree of disruption of the maternal-fetal interface and the likelihood of AFE. However, this suggested relationship is currently a hypothesis-generating observation that must be interpreted prudently. Future studies are needed to clarify whether a pathophysiologic mechanism explains this observation.

Many other risk factors for AFE identified in this study were previously reported by other investigators,^2,4,5,6,8,9,10,11,13,15^ making the results of the current study externally valid. An additional outcome worth highlighting is the association between asthma and AFE. One proposed mechanism for AFE is that it represents an anaphylactoid reaction to fetal antigen in susceptible individuals, such as those with a history of allergies or atopy.^2,13,25,26^ Although the association between asthma and AFE supports this theory, this hypothesis remains to be proven.

### Failure to Rescue

The current study highlights the significant mortality that follows AFE as reported in previous investigations.^2,4,5,6,8,9,10,11,13,15^ When considering the overall maternal mortality rate (0.007% in 2000-2006 and 0.017% in 2016-2017),^1,3,27^ the failure-to-rescue rate after AFE is particularly high (17% overall) and among the highest maternal mortality indicators (Figure 1).

One important finding is that the failure-to-rescue rate after AFE varies markedly in the presence of other clinical factors, ranging from 1.2% to 45.8%. The presence of cardiorespiratory arrest, hypotension and respiratory compromise, and coagulopathy were proposed as key components of the diagnostic criteria for AFE in research studies.^12^ When adhering to these guidelines, the failure-to-rescue rate in our study increases to 38.6% to 45.8% (Figure 2). This estimation is upheld by prior studies^10,15^ that demonstrated higher mortality rates with stricter diagnostic criteria. In addition, use of these guidelines may lead to a diagnostic framework for classic and atypical AFE.^12^ Although maternal mortality rates are significant in both groups, this approach may be useful for both clinical practice and research purposes.

### Incidence

Amniotic fluid embolism was diagnosed in 6 in 100 000 deliveries at the cohort level. When the aforementioned classic criteria (AFE that co-occurred with cardiac arrest and coagulopathy) were applied in a post hoc fashion (Figure 2), the incidence rate decreased to 1.7 per 100 000 deliveries. These statistics are comparable to what has been reported in prior studies^2,4,5,6,7,8,9,10^ (0.7-8.8 per 100 000 deliveries). The incidence rate of AFE can be high when placental abruption occurs in patients with PAS during late-preterm or early-term gestation (eTable 4 in the Supplement), but this clinical scenario is rare, seen in less than 0.1% of the study population. Taken together, these data endorse that AFE is overall an uncommon pregnancy complication.

### Clinical and Research Implications

Because of the rarity of AFE, many health care professionals, particularly trainees, may not be familiar with the disease entity. This gap in knowledge can be further exacerbated by overlapping clinical syndromes, leading to a delay in recognition. The current study expands existing knowledge on the risk factors and mortality of AFE. The high failure-to-rescue rate underscores the urgent need for increased training, such as through simulations, to reinforce a standardized management strategy.^12,28,29^

Several research questions are also raised by the association between PAS and AFE. First, the role of abnormal uterine perfusion as a possible mediator of these 2 pregnancy events warrants further investigation.^30^ Second, whether reduction of the cesarean delivery rate can ultimately reduce the risk of AFE merits further investigation given AFE’s association with PAS.

### Limitations

This study has several limitations. Most importantly, the diagnosis of AFE was based on the *ICD-10* code alone using the administrative-type database without exact medical record review. In addition, the specific diagnostic criteria of AFE in each facility was not available, which may affect the exposure-outcome association. Previous studies^6,31^ suggested that the incidence of AFE appears to be varied across the different diagnostic criteria of AFE. Likewise, accuracy of data for other covariates was not assessable, and this study cannot exclude the possibility of misclassification in an indefinite number of patients, introducing a potential bias of unknown degree.

Unmeasured bias is inherent to this type of study. For example, the chronology between delivery factors and AFE was not available, limiting the ability to determine causality. This limitation particularly applies to interventions such as cesarean and operative deliveries for which the development of AFE may have prompted the need for immediate delivery. This limitation also applies to the chronology of AFE and other SMM indicators. It is therefore unknown whether other indicators occurred at the time of AFE diagnosis or were the consequence of AFE. Neonatal information, health care profressional type, and exact cause of death were also not available. Other factors that are relevant to this subject but were not examined include prolonged stage of labor, placental implantation site other than placenta previa, and delivery anesthesia.

This study only provided the risk factors and mortality outcome, and the effect of therapeutic resuscitation for AFE on failure to rescue was not evaluated. It is unknown whether adherence to the Society for Maternal-Fetal Medicine checklist for initial management of AFE is associated with improved maternal outcome.^28^ Other studies^6,11^ suggested the importance of high-quality cardiopulmonary resuscitation; correction of coagulopathy, including tranexamic acid use; and extracorporeal membrane oxygenation use for AFE management, but these procedures were not assessed in this study. Ascertainment bias is another limitation. In addition to the data-capturing schema, it may be possible that the clinical diagnosis of AFE was made more often in the setting of known risk factors. Finally, generalizability of the results was not assessed and warrants further research.

## Conclusions

This cohort study confirms that AFE is a rare pregnancy complication but is associated with significant maternal outcome. Further research is needed to address the proposed hypotheses as discussed earlier, with particular emphasis on the association between PAS and AFE. Given the rarity of AFE, national and international collaborative efforts would be useful to overcome the type 2 error. Establishing and distributing the universal diagnostic criteria for AFE is also imperative.

## Notes

Supplement. **eFigure.** Temporal Trends of Amniotic Fluid Embolism and the At-Risk Groups **eTable 1.** Study Codes **eTable 2.** Study Demographics **eTable 3.** Extents of Effect Size for Amniotic Fluid Embolism **eTable 4.** A Classification Tree Model for Amniotic Fluid Embolism
