# Supplementary material for: Association of Pregnancy Characteristics and Maternal Mortality With Amniotic Fluid Embolism
Source: JAMA Netw Open. 2022 Nov 18;5(11):e2242842. doi: 10.1001/jamanetworkopen.2022.42842 (PMC9675004; doi:10.1001/jamanetworkopen.2022.42842)
Supplement: Supplement. — eFigure. Temporal Trends of Amniotic Fluid Embolism and the At-Risk Groups eTable 1. Study Codes eTable 2. Study Demographics eTable 3. Extents of Effect Size for Amniotic Fluid Embolism eTable 4. A Classification Tree Model for Amniotic Fluid Embolism [file jamanetwopen-e2242842-s001.pdf]

Supplementary Online Content

Mazza GR, Youssefzadeh AC, Klar M, et al. Association of pregnancy characteristics and maternal mortality with amniotic fluid embolism. *JAMA Netw Open*. 2022;5(11):e2242842. doi:10.1001/jamanetworkopen.2022.42842

eFigure. Temporal Trends of Amniotic Fluid Embolism and the At-Risk Groups

eTable 1. Study Codes

eTable 2. Study Demographics

eTable 3. Extents of Effect Size for Amniotic Fluid Embolism

eTable 4. A Classification Tree Model for Amniotic Fluid Embolism

This supplementary material has been provided by the authors to give readers additional information about their work.

© 2022 Mazza GR et al. *JAMA Network Open.*

eFigure. Temporal Trends of Amniotic Fluid Embolism and the At-Risk Groups

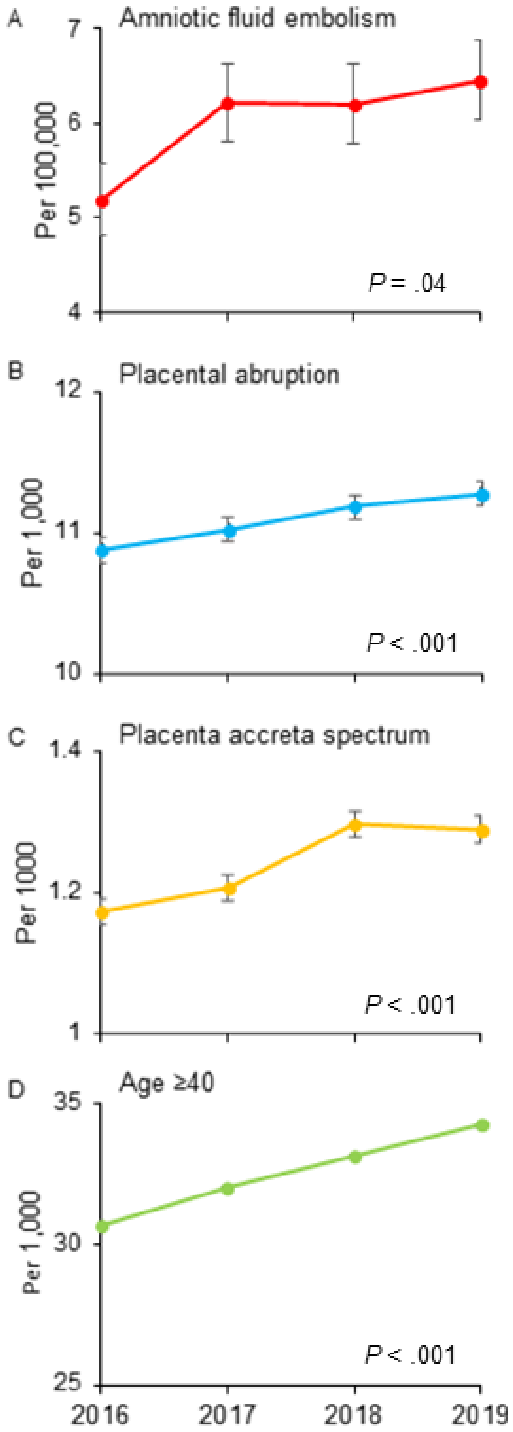

Annual incidence rates with standard error are shown for (A) amniotic fluid embolism, (B) placenta abruption, (C) placenta accreta spectrum, and (D) maternal age ≥40 years. Cochran-Armitage trend test for *P*-values.

© 2022 Mazza GR et al. *JAMA Network Open.*

eTable 1. Study Codes

	Diagnosis-Related Group Codes	ICD-10 CM Codes	ICD-10 PCS Codes
Cesarean delivery	765, 766, 783, 784, 785, 786, 787, 788	O82, O7582	10D00Z0, 10D00Z1, 10D00Z2
Vaginal delivery	767, 768, 774, 775, 796, 797, 798, 805, 806, 807	O80	10D07Z3, 10D07Z4, 10D07Z5, 10D07Z6, 10D07Z7, 10D07Z8
Amniotic fluid embolism		O881	
Hypertensive disorder		O10, O11, O13, O14, O15, O16	
Diabetes mellitus		O24	
Obesity		E660, E661, E662, E668, E669, Z683, Z684, O9921	
Asthma		J45	
Atopic dermatitis		L20	
Allergy status		Z910	
Tobacco use		Z720, O9933, F17, T652	
Alcohol use		F10, O9931, T51, Q860	
Illicit drug use		F11, F12, F13, F14, F15, F16, F18, F190, O9932	
Prior uterine scar		O342	
Uterine myoma		D25, O341	
Grand multiparity		O094, Z641	
Gestational age		Z3A	
Placenta previa		O44	
Placenta abruption		O45	
Placenta accreta spectrum		O432	
Placenta malformation		O431	
Uterine rupture		O710, O711	
Fetal growth restriction		O365, Z364	
Large for gestational age		O366	
Multifetal gestation		O30, O31, O632, O661, Z372, Z373, Z374, Z375, Z376, Z377, O4302	
Fetal breech position		O321, O641	
Fetal anomaly		O35	
Fetal demise		O364	
Polyhydramnios		O40	
Oligohydramnios		O410	
PROM		O42	
Chorioamnionitis		O411	
Vacuum-assisted delivery			10D07Z6 (exclude DRG 770, DRG779, O02, O03, O04, O07, Z332, 10A0)
Forceps delivery			10D07Z3, 10D07Z4, 10D07Z5
Induction labor			3E033VJ, 3E0P7GC, 0U7C7DZ
Manual removal			10D17Z9
Postpartum hemorrhage		O72	

© 2022 Mazza G et al. *JAMA Network Open.*

eTable 2. Study Demographics

Characteristic	No. (%)‡	AFE§	*P*-value
No.	14,648,135 (100)	6.0	
Age (y)			<.001
<25	3,609,892 (24.6)	3.9	
25-29	4,244,822 (29.0)	4.8	
30-34	4,176,197 (28.5)	6.6	
35-39	2,140,779 (14.6)	8.4	
≥40	476,065 (3.3)	16.8	
Unknown	380 (<0.1)	0	
Year			.04
2016	3,756,966 (25.6)	5.2	
2017	3,699,551 (25.3)	6.2	
2018	3,627,394 (24.8)	6.2	
2019	3,564,224 (24.3)	6.5	
Race/ethnicity			<.001
Asian	876,434 (6.0)	10.8	
Black	2,108,915 (14.4)	8.8	
Hispanic	2,911,708 (19.9)	4.5	
White	7,376,209 (50.4)	5.2	
Other*	751,005 (5.1)	6.0	
Unknown	623,864 (4.3)	7.2	
Primary expected payer			<.001
Medicaid	6,231,341 (42.5)	6.3	
Private including HMO	7,509,654 (51.3)	5.6	
Medicare	105,135 (0.7)	14.3	
Self-pay	378,485 (2.6)	4.0	
Others	405,870 (2.8)	8.6	
Unknown	17,650 (0.1)	0.0	
Median household income			.53
QT1 (lowest)	4,072,072 (27.8)	5.9	
QT2	3,656,327 (25.0)	6.4	
QT3	3,601,792 (24.6)	6.1	
QT4 (highest)	3,180,403 (21.7)	5.7	
Unknown	137,540 (0.9)	**	
Hypertensive disorder			<.001
No	12,602,491 (86.0)	5.4	
Pre-gestational	525,650 (3.6)	15.2	
Gestational	764,655 (5.2)	4.6	
Preeclampsia	755,340 (5.2)	11.9	
Diabetes mellitus			.46
No	13,357,975 (91.2)	6.0	
Pre-gestational, type 1	40,430 (0.3)	0	
Pre-gestational, type 2	102,250 (0.7)	**	
Pre-gestational, NOS	14,920 (0.1)	0	
Gestational	1,132,559 (7.7)	6.2	
Obesity			<.001
No	13,048,540 (89.1)	5.7	
Yes	1,599,594 (10.9)	8.1	
Asthma			<.001
No	13,906,985 (94.9)	5.7	
Yes	741,150 (5.1)	11.5	
Atopic dermatitis			.99
No	14,646,565 (>99.9)	6.0	
Yes	1,570 (<0.1)	0	

© 2022 Mazza G et al. *JAMA Network Open.*

Allergy status			.55
No	14,433,660 (98.5)	6.0	
Yes	214,475 (1.5)	7.0	
Tobacco use			.04
No	13,870,875 (94.7)	5.9	
Yes	777,259 (5.3)	7.7	
Illicit substance use			<.001
No	14,259,825 (97.3)	5.8	
Yes	388,310 (2.7)	12.9	
Alcohol use			.64
No	14,626,980 (99.9)	6.0	
Yes	21,155 (0.1)	0.0	
Prior uterine scar			.01
No	12,008,821 (82.0)	5.7	
Yes	2,639,313 (18.0)	7.2	
Uterine myoma			<.001
No	14,444,630 (98.6)	5.9	
Yes	203,505 (1.4)	12.3	
Grand multiparity			<.001
No	14,593,445 (99.6)	6.0	
Yes	54,690 (0.4)	**	
Placenta previa			<.001
No	14,573,945 (99.5)	5.8	
Yes	74,190 (0.5)	40.4	
Placenta malformation			.11
No	14,546,860 (99.3)	6.0	
Yes	101,275 (0.7)	9.9	
Placental abruption			<.001
No	14,485,740 (98.9)	5.5	
Yes	162,395 (1.1)	49.3	
Placenta accreta spectrum			<.001
No	14,629,945 (99.9)	5.8	
Yes	18,190 (0.1)	192.4	
Uterine rupture			<.001
No	14,637,215 (99.9)	5.9	
Yes	10,920 (0.1)	**	
Premature rupture of membrane			.002
No	13,426,715 (91.7)	5.9	
Preterm	390,365 (2.7)	10.2	
Term	831,054 (5.7)	5.4	
Chorioamnionitis			<.001
No	14,305,400 (97.7)	5.8	
Yes	342,735 (2.3)	13.1	
Fetal growth restriction			<.001
No	14,136,560 (96.5)	5.8	
Yes	511,575 (3.5)	11.7	
Large for gestational age			.10
No	14,268,685 (97.4)	6.1	
Yes	379,450 (2.6)	4.0	
Multifetal gestation			<.001
No	14,382,740 (98.2)	5.8	
Yes	265,395 (1.8)	17.0	
Breech presentation			<.001
No	14,082,870 (96.1)	5.9	
Yes	565,265 (3.9)	9.7	
Fetal anomaly			.82

© 2022 Mazza G et al. *JAMA Network Open.*

No	14,493,100 (98.9)	6.0	
Yes	155,035 (1.1)	**	
Polyhydramnios			<.001
No	14,432,550 (98.5)	5.6	
Yes	215,585 (1.5)	30.2	
Oligohydramnios			.08
No	14,256,595 (97.3)	6.1	
Yes	390,660 (2.3)	3.8	
Intrauterine fetal demise			<.001
No	14,533,525 (99.2)	5.8	
Yes	114,610 (0.8)	30.5	
Gestational age (w)			<.001
≥39	9,085,008 (62.0)	4.5	
37-38	3,904,462 (26.7)	5.0	
34-36	1,009,619 (6.9)	9.4	
<34	490,975 (3.4)	18.3	
Unknown	158,070 (1.1)	60.1	
Induction / oxytocin			.98
No	12,229,591 (83.5)	6.0	
Yes	2,418,543 (16.5)	6.0	
Induction / ripening			<.001
No	14,094,220 (96.2)	5.8	
Yes	553,914 (3.8)	10.8	
Induction / dilators			.95
No	14,236,655 (97.2)	6.0	
Yes	411,480 (2.8)	6.1	
Delivery type			<.001
Vaginal	9,341,853 (63.8)	1.8	
Forceps	78,115 (0.5)	6.4	
Vacuum-assisted	499,195 (3.4)	9.0	
Both	815 (<0.1)	0	
Cesarean	4,728,157 (32.3)	14.0	
Manual removal			<.001
No	14,578,570 (99.5)	5.9	
Yes	69,565 (0.5)	21.6	
Hospital bed capacity			.63
Small	2,787,614 (19.0)	5.7	
Mid	4,460,354 (30.4)	6.3	
Large	7,400,167 (50.5)	5.9	
Hospital location / teaching			.03
Rural	1,337,306 (9.1)	5.6	
Urban non-teaching	3,069,382 (21.0)	5.0	
Urban teaching	10,241,446 (69.9)	6.3	
Hospital registry area			<.001
Northeast	2,335,390 (15.9)	5.1	
Midwest	3,089,652 (21.1)	4.5	
South	5,737,373 (39.2)	6.0	
West	3,485,720 (23.8)	7.9	

‡ Number and percentage per column. § Rate per row per 100,000 deliveries. * Including Native American and other grouped by the program. ** Small number suppressed per HCUP guidelines. Abbreviations: AFE, amniotic fluid embolism; QT, quartile; and NOS, not otherwise specified.

© 2022 Mazza G et al. *JAMA Network Open.*

eTable 3. Extents of Effect Size for Amniotic Fluid Embolism

Characteristic	aOR (95%CI)
Placenta accreta spectrum	10.01 (7.03-14.24)
Cesarean delivery	6.41 (5.38-7.64)
Vacuum-assisted delivery	4.95 (3.55-6.88)
Manual placental removal	4.82 (2.86-8.14)
Placental abruption	4.06 (3.17-5.21)
Uterine rupture	3.91 (2.07-7.36)
Polyhydramnios	3.56 (2.76-4.60)
Intrauterine fetal demise	3.32 (2.30-4.79)
Forceps delivery	3.17 (1.30-7.74)
Age ≥40	2.48 (1.86-3.31)
Grand multiparity	2.41 (1.29-4.52)
Induction / ripening	2.19 (1.68-2.86)
Asian individuals	1.83 (1.44-2.31)
West region	1.79 (1.44-2.22)
Chorioamnionitis	1.64 (1.21-2.22)
Asthma	1.59 (1.27-2.00)
Age 35-39	1.57 (1.24-1.98)
Illicit substance use	1.47 (1.09-1.98)
Age 30-34	1.45 (1.17-1.79)
Black individuals	1.41 (1.17-1.70)
Pre-gestational	1.38 (1.08-1.76)
Delivery <34 weeks	1.38 (1.06-1.80)
Fetal growth restriction	1.37 (1.05-1.79)
Pre-eclampsia	1.30 (1.03-1.63)

Effect sizes of independent characteristics associated with amniotic fluid embolism are shown for descending order. The source meta-data is shown in Table 1. Abbreviations: aOR, adjusted-odds ratio; and CI, confidence interval.

© 2022 Mazza G et al. *JAMA Network Open.*

eTable 4. A Classification Tree Model for Amniotic Fluid Embolism

PAS	Abruption	GA	Delivery	Age	Rupture	Polyhydramnios	HTD	(%)*	AFE§
(+)	(+)	34-38						<0.1	6521.7
(+)	(-)						preE	<0.1	**
(-)		34-36			Yes			<0.1	**
(-)		unk		≥40				<0.1	370.4
(-)		<34				Yes		0.1	**
(-)		unk		30-39				0.5	83.4
(+)	(-)						No,other	0.1	**
(-)		unk		<25				0.3	37.3
(-)		<34				No		3.3	15.7
(-)		≥37	CD,Ope					30.8	10.9
(-)		34-36			No			6.8	7.5
(-)		≥37	VD,Ope					57.8	1.2
(-)		unk		25-29†				0.3	0
(+)	(+)	≥39†						<0.1	0

A classification-tree for the incidence rate of AFE was constructed based on the independent characteristics of AFE shown in Table 1. Recursive partitioning analysis with chi-square automatic interaction detector method was used (stopping rule of maximum three layers). The first layer allocator was PAS (192.4 versus 5.8 per 100,000 deliveries, $P < .001$), followed by placental abruption and gestational age in the second layer allocation. * Proportion of identified patter among the study population. § Incidence rate per 100,000 deliveries. † including unknown. ** Small number suppressed per the HCUP guidelines. Abbreviations: PAS, placenta accreta spectrum; Abruption, placental abruption; GA, gestational age; unk, unknown; Rupture, uterine rupture; VD, vaginal delivery; Ope, operative delivery; CD, cesarean delivery; AFE, amniotic fluid embolism; HTD, hypertensive disorder; preE, pre-eclampsia; and other, pre-gestational and gestational hypertensions.

© 2022 Mazza G et al. *JAMA Network Open.*
